# Lactylation-driven gene signatures define breast cancer prognosis: a predictive model and insights into immune microenvironment dynamics

**DOI:** 10.1186/s40001-025-03793-9

**Published:** 2026-01-17

**Authors:** Chao Li, Chao Hu, Xiandong Liu, Ming Li

**Affiliations:** Department of General Surgery, Beijing Luhe Hospital, Capital Medical University, No. 82 Xinhua South Road, Tongzhou District, Beijing, 101100 China

**Keywords:** Breast cancer, Lactylation, Prognostic model, Immune microenvironment, Bioinformatics analysis

## Abstract

**Background:**

Breast cancer is a leading cause of cancer-related mortality worldwide, with poor prognosis largely due to its invasive and metastatic nature. Tumor lactylation plays a crucial role in cancer progression by influencing immune modulation and metabolic reprogramming. This study aimed to identify lactylation-related gene signatures associated with breast cancer prognosis and develop a predictive survival model.

**Methods:**

Bioinformatics analyses were performed using RNA-seq and clinical data from The Cancer Genome Atlas (TCGA) and Gene Expression Omnibus (GEO) datasets. An unsupervised consensus clustering analysis was applied to classify breast cancer samples into distinct groups based on lactylation-related gene expression. Differentially expressed genes (DEGs) between clusters were identified and subjected to functional enrichment analysis. To assess immune-related differences between groups, the ESTIMATE and CIBERSORT algorithms were used, along with an analysis of human leukocyte antigen (HLA) and immune checkpoint molecule expression levels, to explore the relationship between lactylation and the breast cancer immune microenvironment. A prognostic model was constructed using univariate Cox and Lasso regression analyses, followed by validation. Machine learning techniques identified key biomarkers, which were further analyzed for clinical relevance. Additionally, single-cell clustering was performed to investigate the expression patterns of these genes within the breast cancer microenvironment.

**Results:**

Consensus clustering identified two distinct groups: high and low lactylation. Differentially expressed genes were enriched in pathways related to cytokine-cytokine receptor interaction, immune response, cell activation, and adhesion. Lactylation-related genes were found to influence immune cell infiltration in the breast cancer microenvironment. Thirty-seven prognostic lactylation-related genes were identified through univariate Cox regression and used to develop a predictive model. The high-risk group was associated with poorer survival, and the model’s performance was validated in the GEO cohort. Specific hub genes involved in immune modulation and malignant cell proliferation were also identified.

**Conclusion:**

We successfully developed a lactylation-based prognostic model that can assess breast cancer prognosis and potentially guide personalized treatment strategies.

**Supplementary Information:**

The online version contains supplementary material available at 10.1186/s40001-025-03793-9.

## Introduction

Breast cancer is the most common malignant tumor among women globally [1] and ranks as one of the deadliest cancers in women [2]. In China, the number of breast cancer diagnoses has been rising steadily, affecting approximately 300,000 women annually, making it a significant public health concern [3]. While advances in early detection, such as mammography and breast screening programs, have improved survival rates, the prognosis for breast cancer remains highly variable. It often depends on tumor subtype, stage at diagnosis, and individual patient factors [4]. Although treatment options, including surgery, chemotherapy, and targeted therapies, have improved, challenges persist. Some patients may develop resistance to treatment, leading to disease recurrence and metastasis. Furthermore, the heterogeneity of breast cancer complicates treatment decisions, as not all patients respond equally to available therapies [5]. Therefore, there is a pressing need for more accurate prognostic models to guide personalized treatment strategies and improve outcomes for breast cancer patients.

Recent studies have highlighted the role of lactylation, a post-translational modification in which lactate groups are added to lysine residues on proteins, in cancer biology [6, 7]. Lactylation differs from other modifications as it links metabolic shifts within the cell to epigenetic regulation and gene expression (8). In cancer, where metabolic reprogramming is a hallmark, lactate is often overproduced due to aerobic glycolysis, a phenomenon known as the Warburg effect [9]. This excess lactate not only supports tumor growth but also fuels the lactylation of specific proteins that can promote oncogenic processes [10]. Studies suggest that lactylation-related genes are involved in tumor cell proliferation, immune evasion, and metastasis, as they modify proteins that influence chromatin remodeling, transcriptional regulation, and cell signaling pathways crucial for tumor progression [11–14].

In breast cancer, lactylation plays a particularly important role in shaping the tumor microenvironment and influencing cellular behaviors associated with cancer aggressiveness [15, 16]. High lactate levels in breast tumors can create an acidic microenvironment that suppresses immune surveillance and fosters an immunosuppressive state, further promoting tumor progression [17, 18]. Lactylation may also impact signaling pathways that regulate epithelial-mesenchymal transition (EMT), a process by which tumor cells acquire migratory and invasive properties, ultimately leading to metastasis [19]. These findings suggest that lactylation-related genes could serve as potential biomarkers and therapeutic targets in breast cancer, providing new avenues for intervention in disease management.

In this study, we conducted a comprehensive bioinformatics analysis focused on lactylation-related genes to construct a predictive model for breast cancer prognosis and treatment. We performed consensus clustering analysis on the expression matrix of lactylation-related genes, identifying distinct clusters that were closely associated with breast cancer patient outcomes, highlighting the potential prognostic significance of these genes. Following clustering, univariate Cox regression and Lasso regression analyses were applied to select key lactylation-related genes with significant prognostic value. These genes were then used to construct a robust predictive model, which we evaluated for its accuracy and prognostic power in assessing patient survival. Our findings aim to clarify the relationship between lactylation modifications and breast cancer, thereby enhancing our understanding of tumor behavior and paving the way for more personalized therapeutic approaches. We present this article in accordance with the TRIPOD reporting checklist.

## Materials and methods

### Data collection and processing

Breast cancer (BRCA) RNA-seq transcriptome data and corresponding clinical information were retrieved from The Cancer Genome Atlas (TCGA, https://cancergenome.nih.gov/) and used as the training cohort for constructing the prognostic model. To ensure the robustness of the survival analysis, cases without survival data were excluded. The data were downloaded in fragments per kilobase million (FPKM) format to maintain consistency in subsequent analyses. Additionally, the GSE131769 dataset from the Gene Expression Omnibus (GEO, http://www.ncbi.nlm.nih.gov/geo), which includes 301 breast cancer samples with available survival time and outcomes, was employed as the validation cohort. Batch effect correction between the TCGA and GEO datasets was performed using the ComBat function from the "sva" R package to ensure comparability of expression profiles. Based on existing literature, we compiled a limited set of 332 genes associated with lactylation, a post-translational modification involving the covalent attachment of lactate to proteins, for further investigation. [20]. The study was conducted in accordance with the Declaration of Helsinki (as revised in 2013).

### Unsupervised clustering analysis

An unsupervised consensus clustering analysis was conducted on the lactylation-related gene set to classify BRCA patients into molecular subtypes. The R package "ConsensusClusterPlus" was used to determine the optimal number of clusters, with clustering repeated 100 times and a pItem value of 0.8 to assess cluster stability. The optimal number of clusters was identified as 2, resulting in the division of patients into two groups. Kaplan–Meier survival analysis and the log-rank test were then performed to compare overall survival between the clusters.

### Differentially expressed lactylation-related genes between clustered groups

After performing consensus clustering on BRCA samples, we used the R package “limma” to identify differentially expressed lactylation-related genes between the two resulting groups. A threshold of |logFC|> 0 and p-value < 0.05 was applied to select genes with significant expression differences between the clusters.

### Identification of differentially expressed genes (DEGs) and functional enrichment analysis

Differentially expressed genes (DEGs) between the clusters were identified, and functional enrichment analyses were performed using the R package “clusterProfiler.” Enrichment analysis was conducted for Gene Ontology (GO) categories, including Biological Process (BP), Cellular Component (CC), and Molecular Function (MF). Additionally, Kyoto Encyclopedia of Genes and Genomes (KEGG) pathways and Gene Set Enrichment Analysis (GSEA) were performed to identify relevant pathways, using the “c5.go.symbols.gmt” and “c2.cp.kegg.symbols.gmt” databases.

### Analysis of tumor immune microenvironment across clusters

To characterize the immune and stromal components of the breast cancer tumor microenvironment, we first computed ESTIMATE (Estimation of STromal and Immune cells in MAlignant Tumor tissues using Expression data) scores, immune scores, stromal scores, and tumor purity across clusters using the ESTIMATE algorithm. Next, we applied the CIBERSORT (Cell-type Identification By Estimating Relative Subsets Of RNA Transcripts) algorithm to analyze the distribution of 22 immune cell types within TCGA-BRCA samples. Finally, one-way ANOVA was performed to compare the expression levels of Human Leukocyte Antigen (HLA) genes and immune checkpoint molecules across the clusters.

### Development and validation of a prognostic model based on lactylation-related genes

A lactylation-based risk model was constructed by first identifying significant prognostic genes using univariate Cox regression analysis. Lasso regression with tenfold cross-validation was then applied to determine the optimal penalty parameter (λ) and refine the model [21], generating a risk score that stratified patients into high- and low-risk groups based on the median score. Referring to the previously published study, we assessed the predictive accuracy of the model using Kaplan–Meier survival analysis and risk score distribution plots [22]. The model’s performance was further validated using an independent GEO dataset.

### Identification of key lactylation-related genes via machine learning

Machine learning approaches were used to identify key hub genes associated with prognosis. Using random forest (RF) and support vector machine (SVM) algorithms from the “randomForest” and “e1071” R packages, genes consistently identified across Lasso, RF, and SVM were designated as core biomarkers.

### Examination of hub gene expression, clinical relevance, and microenvironmental distribution

Wilcoxon’s test was applied to compare hub gene expression levels between tumor and normal tissues, followed by correlation analyses with clinical characteristics, immune cell infiltration, immune checkpoints expression, and functional pathways. Single-cell RNA sequencing data were also used to map the expression distribution of key genes across cell types in the BRCA tumor microenvironment.

### Tissue sample acquisition and preparation

From June 2024 to December 2024, breast tissue samples, including both tumor and adjacent normal tissues, were collected from 10 newly diagnosed breast cancer patients at the General Surgery Department of Beijing Luhe Hospital. These samples were used for molecular analyses to study gene expression profiles in breast cancer. Before sample collection, all participants provided written informed consent, and the study protocol was approved by the institutional ethics committee. The samples were processed and stored under standardized conditions to ensure RNA integrity for reliable qPCR analysis.

### RNA extraction and qPCR gene expression analysis

RNA extraction from fresh tissue samples was carried out using the RNA isolater Total RNA Extraction Reagent (Vazyme, Nanjing, China), strictly following the manufacturer’s protocol. RNA concentration was assessed with a NanoDrop 2000 spectrophotometer (Thermo Scientific, Wilmington, DE, USA). Next, 1 µg of total RNA was reverse-transcribed into cDNA using HiScript II Q Select RT SuperMix (Vazyme, Nanjing, China), as per the recommended procedure. Quantitative PCR analysis was conducted with SYBRGreen qPCRMix (Biosharp, Beijing, China) on a CFX96 Touch system (BioRad, Mississauga, ON, Canada). Target gene Ct values were normalized to β-Actin, and relative expression levels were computed using the 2^−ΔΔCt^ method. The primer sequences employed included β-Actin (forward: 5′‐GAAGAGCTACGAGCTGCCTGA‐3′, reverse: 5′‐CAGACAGCACTGTGTTGGCG‐3′) and RPL14 (forward: 5′‐AACACTTTCGATTCCACCTTGG‐3′, reverse: 5′‐TTGTGGGTACATTTGTGGTACAG‐3′).

### Statistical analysis

All analyses were conducted using R version 4.4.0. Statistical comparisons between two groups were performed using either Wilcoxon rank-sum test for nonparametric data or Student’s t-test for parametric data. One-way ANOVA was applied for comparisons across multiple groups. Correlation analyses used Pearson’s or Spearman’s tests, as appropriate. Survival analysis was conducted using Kaplan–Meier plots with significance evaluated by the Log-rank test, and a p-value < 0.05 was considered significant.

## Results

### Identification of DEGs and lactylation-based clustering

**Fig. 1:**
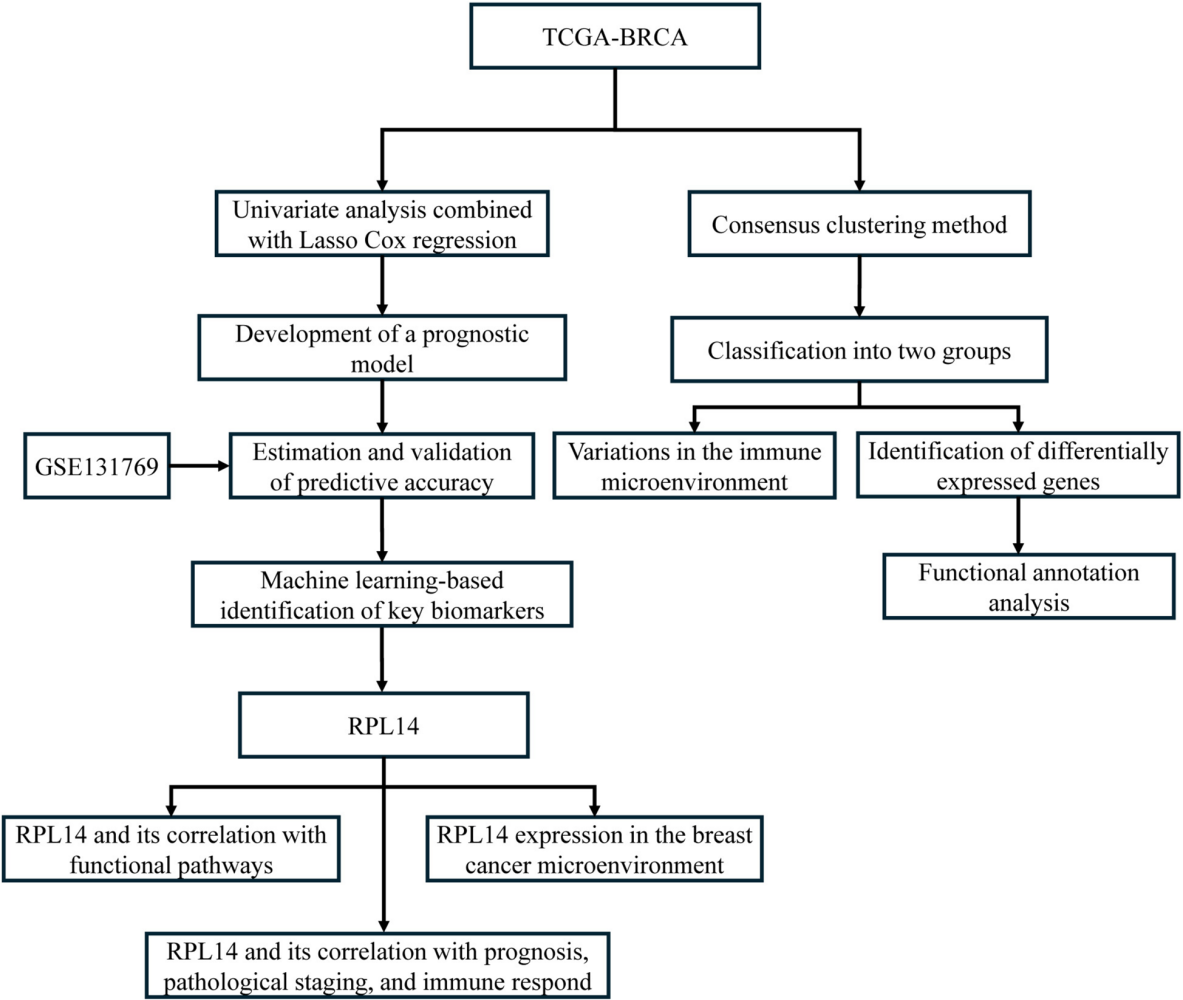
The schematic representation of the study workflow

**Fig. 2:**
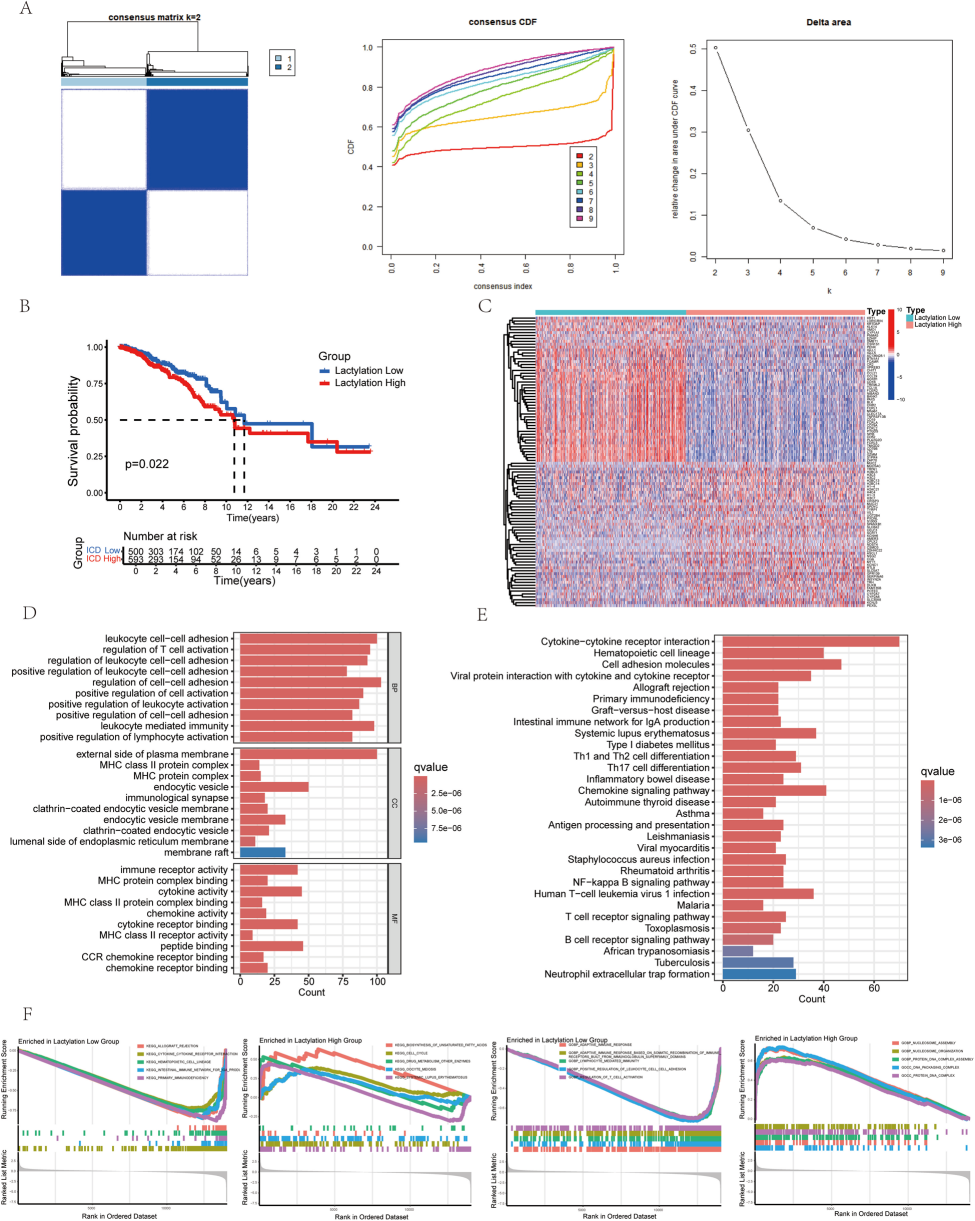
Breast Cancer Patients Were Categorized into Two Clusters Based on the Expression Profiles of Lactylation-Related Genes. **A** Consensus clustering analysis was performed on 1097 TCGA-BRCA samples, dividing them into two groups based on the expression of 332 lactylation-related genes. **B** Kaplan–Meier survival analysis demonstrates the survival differences between the two clusters. **C** Heatmap visualizes the expression patterns of differentially expressed genes (DEGs) between the two groups. **D** Gene Ontology (GO) enrichment analysis of DEGs in the high- and low-risk groups. **E** Kyoto Encyclopedia of Genes and Genomes (KEGG) pathway enrichment analysis of DEGs between the groups. **F** Gene Set Enrichment Analysis (GSEA) reveals enriched pathways associated with the high- and low-risk groups

The study workflow is outlined in Fig. 1. We performed clustering analysis on TCGA-BRCA samples using lactylation-related gene expression profiles. Optimal clustering at k = 2 resulted in two distinct groups: a high lactylation expression group (Lactylation High Group, C1) and a low lactylation expression group (Lactylation Low Group, C2) (Fig. 2A). Kaplan–Meier survival analysis indicated that the Lactylation High Group had a significantly poorer prognosis than the Lactylation Low Group (Fig. 2B). A heatmap displayed the expression patterns between these groups (Fig. 2C).

### Functional enrichment analysis of lactylation-based clusters

We performed GO, KEGG, and GSEA analyses to explore the functional implications of DEGs between the lactylation clusters. GO analysis showed significant enrichment in Biological Process pathways related to immune regulation, including regulation of T-cell activation, positive regulation of leukocyte activation, leukocyte mediated immunity, and positive regulation of lymphocyte activation (Fig. 2D). Cellular Component enrichment highlighted immune-related structures such as MHC class II protein complex, immunological synapse, and endocytic vesicle (Fig. 2D). Molecular Function analysis revealed associations with immune receptor activity, MHC protein complex binding, cytokine activity, and chemokine receptor binding (Fig. 2D).

KEGG pathway analysis indicated key pathways, including immune regulation pathways (e.g., cytokine-cytokine receptor interaction, hematopoietic cell lineage), autoimmune and allergic disease pathways (e.g., systemic lupus erythematosus, type I diabetes mellitus), transplant-related immune response pathways (e.g., allograft rejection, graft-versus-host disease), and T-cell differentiation and inflammation pathways (e.g., Th1 and Th2 cell differentiation, inflammatory bowel disease) (Fig. 2E).

We compared the differences between high and low lactylation risk groups using the “c5.go.symbols.gmt” and “c2.cp.kegg.symbols.gmt” databases. The low-risk group was enriched in immune-related KEGG pathways (e.g., cytokine-cytokine receptor interaction, immune network for IgA production) and adaptive immune response GO terms (e.g., T-cell activation, lymphocyte-mediated immunity) (Fig. 2F). In contrast, the high-risk group was enriched in pathways related to biosynthesis and cell cycle regulation (e.g., unsaturated fatty acid biosynthesis, cell cycle) as well as chromatin and nucleosome organization (e.g., nucleosome assembly, DNA packaging complex) (Fig. 2F). These analyses suggest that lactylation-related genes may play a role in modulating breast cancer microenvironment and metabolism.

### Immune landscape analysis between lactylation clusters

**Fig. 3:**
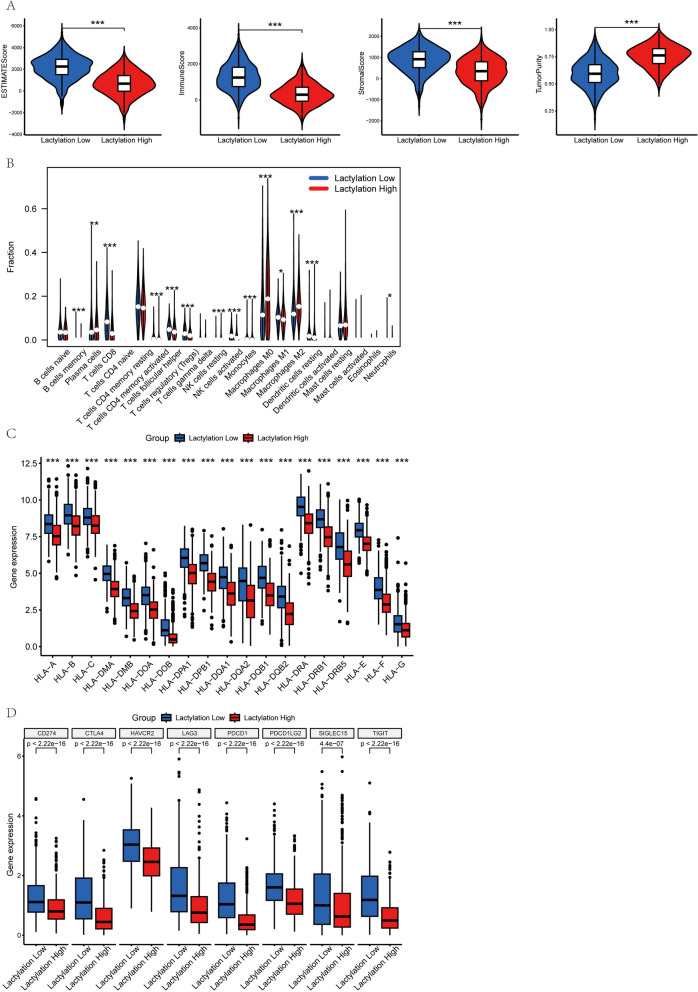
Analyzing immune microenvironment differences in breast cancer between the lactylation high and low groups. **A** Box plots depict variations in ESTIMATE score, immune score, stromal score, and tumor burden between the two groups. **B** Differences in the infiltration levels of 22 immune cell types between the groups. **C** Differential expression of human leukocyte antigen (HLA) genes between the high and low groups. **D** Expression differences in immune checkpoint genes across the groups. Statistical significance is represented as follows: “*” for P < 0.05, “**” for P < 0.01, “***” for P < 0.001, and “ns” for no significance

The ESTIMATE algorithm revealed that the Lactylation High Group had lower ESTIMATE (p < 0.001), immune (p < 0.001), and stromal scores (p < 0.001), while tumor purity (p < 0.001) was higher than in the Lactylation Low Group (Fig. 3A). CIBERSORT analysis indicated that the Lactylation High Group exhibited lower levels of CD8 + T cells, NK cells activated, and macrophage M1 but higher levels of macrophage M2 (Fig. 3B), underscoring the potential role of lactylation in shaping the breast cancer immune microenvironment. Differences in Human Leukocyte Antigen (HLA) expression and immune checkpoint molecules were also observed between the two groups (Fig. 3C and D).

### Construction of lactylation-related prognostic model

**Fig. 4:**
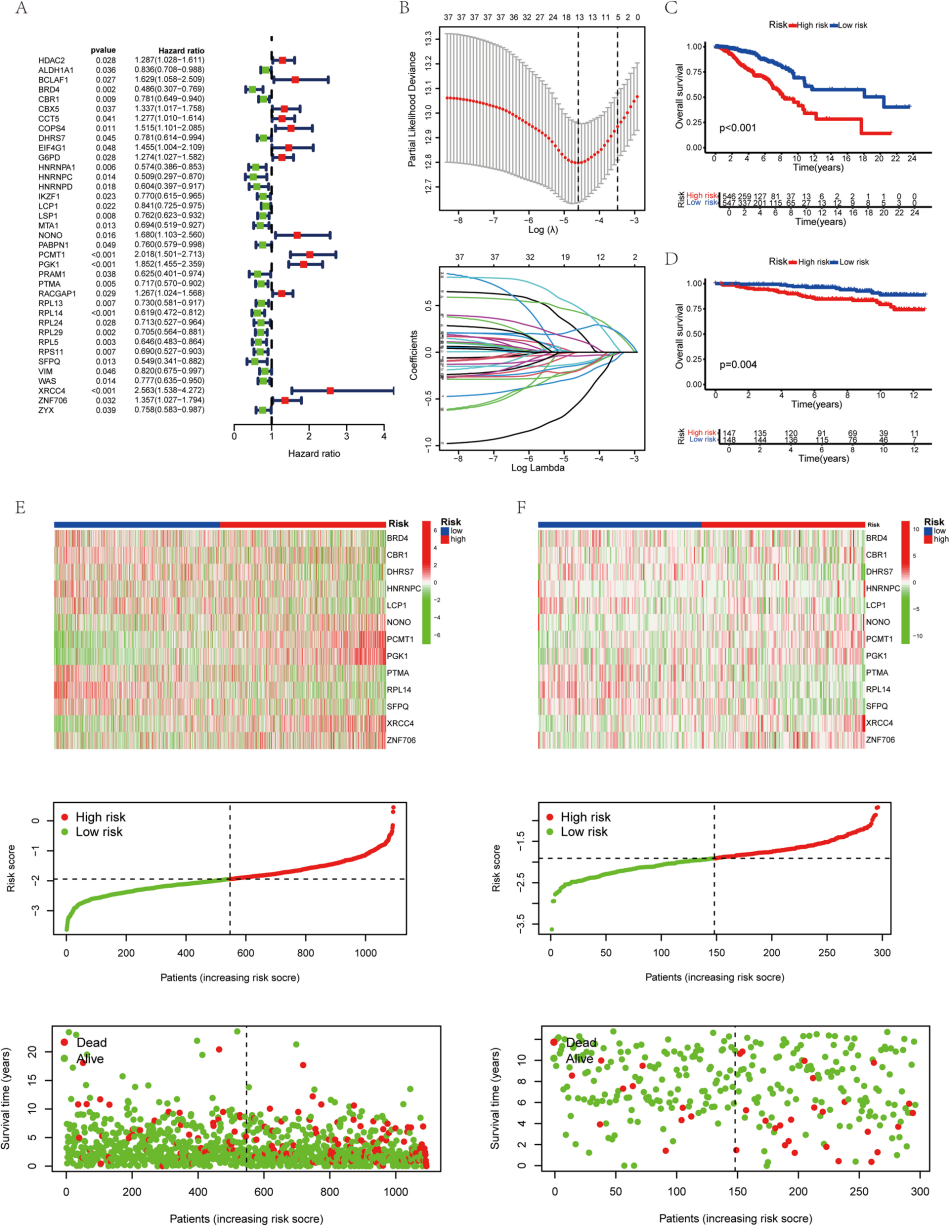
Development and Validation of a 13-Gene Prognostic Model (Lactyscore) for Breast Cancer. **A** Univariate Cox regression analysis identifies 37 genes linked to the prognosis of breast cancer patients. **B** Lasso Cox regression analysis establishes a 13-gene prognostic model called Lactyscore. **C** Kaplan–Meier survival curves comparing survival outcomes between high- and low-risk groups based on The Cancer Genome Atlas (TCGA). **D** Kaplan–Meier survival curves showing survival differences between high- and low-risk groups using the Gene Expression Omnibus (GEO) database. **E** Heatmap illustrating the expression of 13 genes identified by Lasso Cox regression in high- and low-risk groups from TCGA data. **F** Heatmap displaying the expression of the same 13 genes, along with the distribution of risk scores and survival status, in high- and low-risk groups from the GEO dataset. The symbols are defined as follows: “***” signifies P < 0.001

Univariate Cox regression analysis identified 37 lactylation-related genes with significant associations with patient prognosis (Fig. 4A). Lasso regression was then applied to refine these genes, resulting in a 13-gene risk model named LactyScore (Fig. 4B). Kaplan–Meier curves confirmed that the high-risk group, as defined by LactyScore, had significantly worse outcomes in both the training (TCGA dataset, Figs. 4C) and validation datasets (GEO dataset, Figs. 4D). The risk score distribution further supported LactyScore as a robust indicator for BRCA prognosis, with TCGA data shown in Fig. 4E and GEO data in Fig. 4F.

**Fig. 5:**
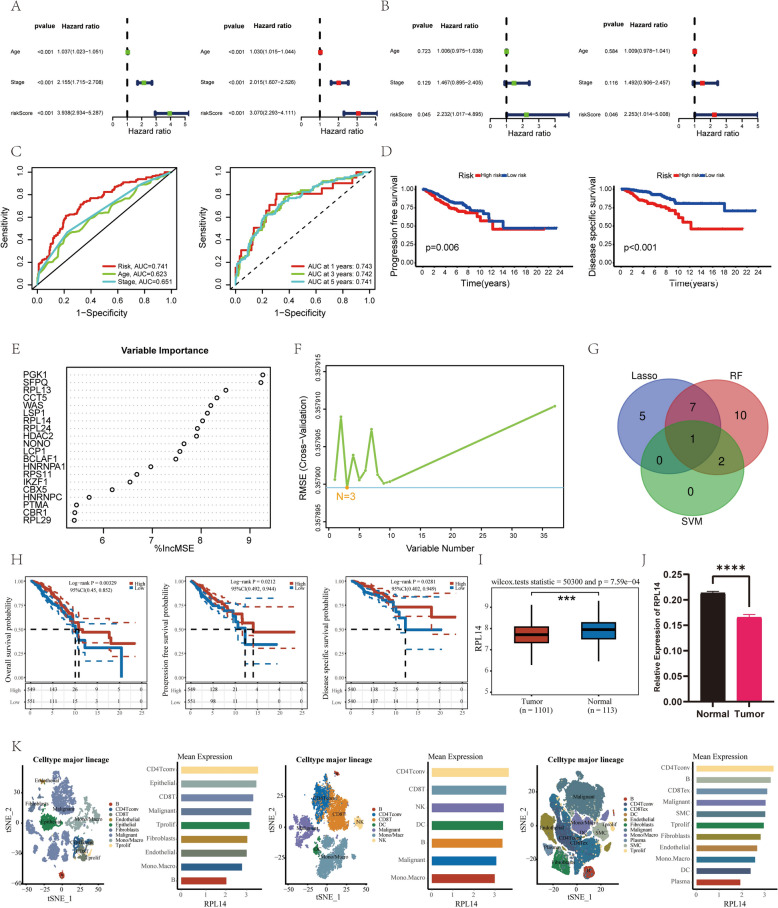
Comprehensive Prognostic Analysis of Breast Cancer Using TCGA and GEO Datasets. (**A**) and (**B**) Univariate and multivariate Cox regression analyses based on the TCGA and GEO datasets, respectively. **C** ROC curves based on the TCGA dataset: left, for age, TNM stage, and LactyScore; right, for 1-, 3-, and 5-year predictions of LactyScore. **D** Kaplan–Meier survival curves based on the TCGA dataset: left, progression-free survival (PFS); right, disease-specific survival (DSS), evaluating the significance of LactyScore. **E** Random forest analysis for gene selection. **F** Support vector machine (SVM) analysis for gene identification. **G** Intersection of genes identified by Lasso regression, random forest, and SVM analyses. **H** Kaplan–Meier survival curves illustrating the association between RPL14 expression and survival outcomes in breast cancer patients: left, overall survival (OS); middle, progression-free survival (PFS); right, disease-specific survival (DSS). **I** Box plot showing the differential expression of RPL14 between breast cancer tissues and adjacent normal tissues. **J** qPCR analysis showing the differential expression of RPL14 between breast cancer tissues and adjacent normal tissues. **K** Expression distribution of the RPL14 gene in the breast cancer microenvironment based on single-cell datasets: left, GSE148673; middle, GSE150660; right, GSE176078

Based on the TCGA database, we found that Age, TNM staging, and LactyScore were all significant prognostic factors in both univariate and multivariate Cox regression analyses (Fig. 5A). However, in the GEO dataset, only LactyScore was found to be a significant prognostic factor, further highlighting its potential as a key predictor of patient outcomes (Fig. 5B). ROC curves were plotted for age, TNM staging, and LactyScore, with the areas under the curves (AUCs) being 0.623, 0.651, and 0.741, respectively (Fig. 5C). Additionally, ROC curves for LactyScore at 1-year, 3-year, and 5-year intervals were also plotted, with the AUCs being 0.743, 0.742, and 0.741, respectively (Fig. 5C).

We observed that the high-risk group exhibited a poorer prognosis compared to the low-risk group in both Progression-Free Survival (PFS) (Fig. 5D) and Disease-Specific Survival (DSS) (Fig. 5D), based on the TCGA-LIHC database. Furthermore, we constructed nomograms incorporating Age, TNM staging, and LactyScore as potential tools for assessing patient prognosis. These nomograms were designed to predict Overall Survival (OS) (Supplementary Fig.  1 A), PFS (Supplementary Fig. 1B), and DSS (Supplementary Fig.  1 C), with corresponding concordance indices (C-indices) calculated to evaluate their predictive accuracy.

### Identification and characterization of RPL14 as a prognostic biomarker in cancer

To identify key biomarkers for cancer prognosis, we employed a combination of advanced machine learning techniques, including Lasso regression, Random Forest (Fig. 5E), and Support Vector Machine (Fig. 5F). Through this multi-faceted analysis, we pinpointed RPL14 (Fig. 5G) as a core gene of interest and evaluated its association with critical prognostic indicators such as Overall Survival (OS), Progression-Free Survival (PFS), and Disease-Specific Survival (DSS). Our results demonstrated that higher expression levels of RPL14 were significantly associated with better patient prognosis, particularly in terms of OS (Fig. 5H), PFS (Fig. 5H), and DSS (Fig. 5H), underscoring its potential as a promising biomarker with important clinical implications for cancer prognosis.

Further investigation revealed a marked differential expression of RPL14 between breast cancer tissues and adjacent normal tissues, with RPL14 expression notably higher in the normal tissues based on TCGA database (p-value = 7.59e-04) (Fig. 5I). Through qPCR validation using clinically collected breast cancer tissues and adjacent normal tissues, we also found that the expression level of RPL14 was significantly higher in adjacent normal tissues (Fig. 5J). This differential expression was also linked to various clinical parameters, including tumor stage (T stage), lymph node status (N stage), metastatic status (M stage), and overall TNM staging (Supplementary Fig.  2 A). Specifically, RPL14 expression was found to be significantly reduced in advanced T and N stages, as well as in the M1 stage, compared to normal tissues, with these differences showing strong statistical significance. Additionally, the expression level of RPL14 was found to correlate with the proportion of immune cell infiltration (Supplementary Fig. 2B) and the expression levels of immune checkpoint-related molecules in the breast cancer immune microenvironment (Supplementary Fig.  2 C).

In addition, we explored the relationship between RPL14 expression and pathway activity, finding a negative correlation between RPL14 expression and tumor cell proliferation. This was evident in the correlation analysis, where higher RPL14 expression was inversely associated with three key pathways: DNA replication (Correlation coefficient = − 8.57e-02, P-value = 4.66e-03) (Supplementary Fig. 2D), G2/M checkpoint (Correlation coefficient = − 2.25e-01, P-value = 7.55e-14) (Supplementary Fig. 2E), and the tumor proliferation signature (Correlation coefficient = − 1.89e-01, P-value = 3.88e-10) (Supplementary Fig. 2). These findings suggest that RPL14 may play a role in modulating key cellular processes related to tumor progression, such as cell cycle regulation and proliferation.

Finally, using single-cell RNA sequencing data from three independent datasets-GSE148763 (Fig. 5K), GSE150660 (Fig. 5K), and GSE176078 (Fig. 5K)-we conducted a detailed examination of RPL14 expression across diverse cell types within the tumor microenvironment. The results revealed that RPL14 was predominantly expressed in tumor-associated immune cells and malignant cells, suggesting its potential involvement in the modulation of both the immune response and tumor progression. These findings emphasize the critical role of RPL14 in the complex interactions that define the cancerous microenvironment and highlight its potential as a therapeutic target or prognostic biomarker in cancer immunology.

## Discussion

In this study, we developed a robust prognostic model for breast cancer based on lactylation modification-related genes. By systematically analyzing their expression, we effectively stratified patients by risk and accurately predicted survival outcomes. Additionally, the Lactyscore was instrumental in evaluating the immune landscape, providing a promising tool for personalized treatment strategies in breast cancer.

Breast cancer, one of the most common and heterogeneous malignancies, is characterized by its variable prognosis and treatment response. Recent studies have emphasized the significance of lactylation in regulating cancer cell metabolism, gene expression, and tumor progression [23, 24]. Additionally, this modification plays a critical role in shaping the tumor microenvironment and modulating immune cell function, potentially influencing tumor progression and metastasis [25]. Therefore, understanding the expression patterns and mechanisms of lactylation-related genes could improve our understanding of breast cancer prognosis and lead to new therapeutic approaches.

By performing consistency cluster analysis, we observed distinct differences in overall survival, immune cell infiltration, and pathway enrichment between these two groups. Univariate regression identified several lactylation-related genes as potential prognostic indicators for breast cancer, and Lasso analysis further selected key genes to construct the LactyScore. External datasets validated the model’s robust prognostic performance and high accuracy in predicting patient survival. Lactyscore, as an independent prognostic indicator, was validated through both univariate and multivariate Cox regression analysis in two independent datasets, TCGA and GEO. The results confirmed that Lactyscore could serve as an independent prognostic factor in both cohorts. Furthermore, for survival-related endpoints such as overall survival (OS), progression-free survival (PFS), and disease-specific survival (DSS), the development of nomograms based on Lactyscore will aid clinicians in providing more accurate prognostic assessments, facilitating better individualized patient management and decision-making in clinical settings.

Through machine learning, RPL14 was pinpointed as a key gene potentially acting as a protective factor with clinical significance. Its expression is notably reduced in breast cancer tissues compared to adjacent normal tissues, and the downregulation of RPL14 correlates with poor survival outcomes in patients, which is consistent with previous research finding [26]. Research has indicated that RPL14 is linked to nasopharyngeal carcinoma, where it has been found to inhibit key processes such as proliferation, migration, invasion, and epithelial-mesenchymal transition (EMT) [27]. These findings imply that RPL14 may function as a tumor suppressor in specific contexts by restraining cancer progression.

Furthermore, RPL14 appears to influence immune cell infiltration and the expression of immune checkpoint markers, highlighting its potential therapeutic significance [28, 29]. The immune microenvironment in breast cancer is highly dynamic and plays a critical role in the efficacy of various therapeutic interventions, particularly immunotherapies [30, 31]. While immune checkpoint inhibitors (ICIs) have improved breast cancer treatment, but response is limited by tumor immune evasion, such as checkpoint upregulation and microenvironmental immunosuppression [32, 33]. A study has shown RPL14 expression may be linked to the infiltration of immune cells and immune responses within the tumor microenvironment [34].

More importantly, we discovered that RPL14 impacts patient prognosis through its regulation of pathways associated with malignant tumor cell proliferation. Specifically, we observed that RPL14 expression is negatively correlated with genes involved in DNA replication, G2/M checkpoint regulation, and tumor proliferation signatures, which are critical for cell cycle progression and uncontrolled tumor growth. Research also suggests that RPL14 may influence tumor cell proliferation by modulating key processes, including the cell cycle, DNA repair, and protein synthesis [35]. RPL14's downregulation may accelerate tumor progression by affecting these pathways [27]. Moreover, RPL14 has demonstrated anti-tumor properties in cervical cancer by inhibiting cell proliferation and migration [36]. Additional research has shown that RPL14 correlates with cisplatin resistance in breast cancer and modulates signaling pathways affecting cancer cell proliferation and apoptosis [37].

Furthermore, single-cell sequencing data revealed that RPL14 is primarily expressed on the surface of both immune cells and malignant proliferative cells, suggesting its dual role in regulating tumor proliferation and modulating the immune microenvironment. This dual involvement highlights RPL14’s potential as a prognostic biomarker and therapeutic target, influencing both tumor growth and immune response. In summary, the effects of RPL14 on cell cycle regulation, tumor proliferation, and immune cell function underscore its clinical relevance in breast cancer, with implications for improving patient outcomes and predicting responses to immune therapies.

Overall, our study highlights the prognostic potential of lactylation-related genes in breast cancer. By integrating bioinformatics and machine learning techniques, we developed the Lactyscore model, which effectively stratifies patients into high- and low-risk groups with strong associations to overall survival. However, it is important to note that our conclusions are based on retrospective analyses of publicly available datasets, which inherently carry biases, such as patient selection and data quality variability. Although we validated the model using TCGA and GEO cohorts, further confirmation in independent, prospective cohorts is needed to ensure its robustness and generalizability. Additionally, while we identified key lactylation-related genes, their functional roles in predicting breast cancer prognosis require further investigation. The lack of direct in vitro and in vivo validation limits our understanding of their biological mechanisms. Therefore, additional experimental studies are necessary to confirm their involvement in tumor progression. Validation in patient-derived samples would further strengthen the clinical relevance of these findings. Lastly, the molecular mechanisms by which lactylation-related genes influence breast cancer progression remain unclear and warrant further investigation, which may reveal novel therapeutic targets to improve patient outcomes.

## Conclusion

Lactylation modification-related genes represent promising biomarkers for predicting prognosis and therapeutic response in breast cancer. The validated LactyScore model offers a robust tool for risk stratification by linking patient survival outcomes. Our findings highlight the critical role of lactylation in shaping the tumor microenvironment and driving cancer progression, suggesting new opportunities for personalized treatment. Future research should further clarify the molecular mechanisms of lactylation and explore its potential as a therapeutic target.

## Supplementary Information

Supplementary file 1. Figure 1. Comprehensive Analysis of RPL14 in Breast Cancer: Prognostic Value, Expression Patterns, and Immune Microenvironment Correlations.,, andNomograms for predicting OS, PFS, and DSS of breast cancer patients based on the TCGA dataset, along with corresponding C-index evaluations

Supplementary file 2. Figure 2. Associations of RPL14 Expression with Clinicopathological Features, Immune Microenvironment, and Functional Pathways in Breast Cancer.Relationship between RPL14 expression and pathological stages of breast cancer based on the TCGA dataset.Correlation between RPL14 expression and immune cell infiltration in the breast cancer immune microenvironment.Correlation between RPL14 expression and the expression levels of immune checkpoint-related genes in the breast cancer immune microenvironment.,, andCorrelation between RPL14 expression and the DNA replication pathway, G2M checkpoint pathway, and tumor proliferation signature pathway, respectively

## Data Availability

No datasets were generated or analysed during the current study.
